# The Cost of Operating: Analysis of Single-Use Instrument Costs for Craniotomies

**DOI:** 10.7759/cureus.43099

**Published:** 2023-08-07

**Authors:** Uttam Kalluri, Lauren Stone, Jeffrey A Steinberg

**Affiliations:** 1 Department of Neurological Surgery, University of California (UC) San Diego, San Diego, USA

**Keywords:** operative costs, surgical instruments, quality improvement, hospital costs, craniotomy

## Abstract

Introduction

All-cause craniotomies comprise a significant portion of neurosurgical practice as well as hospital costs. While some instruments are reusable with a fixed cost, price variability for similar single-use instruments exists. A better understanding of these cost variations within cranial procedures can better inform operating physicians to be cost-sensitive stewards.

Objective

In this study, we examine how single-use items contribute to the overall cost of cranial procedures.

Methods

A de-identified institutional database containing records of all single-use items from craniotomies between July 1, 2019, and June 30, 2020, was subject to a longitudinal analysis by three independent parties (one senior surgeon, one resident, and one medical student). Four hundred and sixty-nine unique single-use items were identified and classified by function. Similar items were combined, and a range of costs was provided. Three sample cases with sum costs were reviewed for cost division and primary contributors.

Results

The category with the highest median cost across all cases was non-specialty implants comprising dural onlays, mesh, aneurysm clips, and plates. The category with the lowest median cost was personal protective equipment. The items with the most cost variability were sterile surgical patties due to the variety of sizes and preset multipacks. The proportion of cost generators varies from craniotomy indication.

Conclusion

While institution dependent, awareness of cost generators in cranial cases is important for economic stewardship. For single-use items, costs are highly variable and not insignificant. Surgeons and neurosurgical departments are responsible for allocating single-use items in a responsible and efficient manner.

## Introduction

Surgical costs constitute some of the greatest medical center expenses across the country with more than $175 billion spent on operating room procedures in 2011 alone [1]. Neurosurgical operating costs have increased correspondingly with craniotomies in particular comprising some of the most expensive operative procedures [2-4]. While some operative costs are fixed/standing, variation exists for one-time-use items, which are utilized in a case-dependent manner according to the hospital’s purchased stock and the surgeon’s preference. How these items ultimately aggregate to total cost and to what degree this varies by craniotomy type is unknown. In this study, we analyze the cost of single-use surgical items during cranial procedures to evaluate use patterns as well as variation between case types.

Critically, our article’s purpose is to capture a year-long snapshot of the costs associated with single-use instruments at our institution. The purpose of this article is not to examine how durable instruments contribute to the total cost of a craniotomy procedure, how the costs of single-use instruments of outside institutions compare to ours, or how many of our single-use instruments contribute to operative waste. Each of these questions is beyond the scope of this article, which is to make neurosurgeons aware of single-use-item costs associated with craniotomies. This article was previously presented as a meeting abstract at the 2022 Congress of Neurological Surgeons Annual Meeting on October 8-12, 2022.

## Materials and methods

A retrospective longitudinal review was performed of a de-identified institutional costs database at a single institution between July 1, 2019, and June 30, 2020. Craniotomies from all attending surgeons were identified using the keywords “Craniotomy,” “Craniotomy, with Image Guidance,” and “Surgical Exposure Craniotomy.” Craniotomies used for deep brain stimulation were not included in this study, as the items used therein are not representative of the most commonly used single-use items in our institution's neurosurgical cases. The resulting list was delimited to single-use items and the cost/range of costs and use frequency for each item were electronically tallied. Items missing key data points such as individual cost or frequency of utilization were excluded. For items with more than one cost (usually small changes in price across the analyzed time frame), the use frequency of each item at a given cost was evaluated. If even for both listed costs, the average was taken. If the use was weighted heavily toward one cost, that cost was listed. 

All three authors independently categorized the items into salient categories, which were then compared and culled to the following: drains, drill items, hemostatic agents, hemostatic instruments, specialty implants, non-specialty implants, personal protective equipment (PPE), sutures, and miscellaneous items. 

Three sample surgical craniotomies - epidural hematoma evacuation, supratentorial parenchymal brain tumor resection, and suboccipital craniectomy with C1 laminectomy - were then extracted from the database after subjecting it to simple random sampling and delimited to the above single-use items only. The overall cost of the case and single-use-item breakdown were then tabulated. 

The provided data source was de-identified from its inception, which exempted this study from our university’s Institutional Board Review approval. All activities within this study have been performed in accordance with our university’s research ethics policies. 

## Results

Four hundred and sixty-nine unique items were identified and included in this study. Non-specialty implants were the most expensive items ($53-$3643) with the widest intra-item variation of the entire set ($431.88-$1147.79) attributed to dural onlays. Most of these implants were used once per case. PPE was the least expensive category when controlled for unit cost and frequency of use ($1.88-$3.50) (Table 1).

**Table 1:** Single-Use Craniotomy Items EVD, external ventricular drain.

Subcategories and Items	Cost, in Dollars
Drapes	9.80-143.26
Fluoroscopy	29.95
Microscope	9.80-143.26
Hemostatic Instruments and Agents	1.38-537.00
Bipolar Forceps	55.48-537.00
Monopolar	132.35-134.08
Hemostatic Matrix Sealant (2mL-4 mL)	53.00-229.99
Sterile Surgical Patties	1.38-3.66; 9.95-259.84
Regular (1/2 X 3”-1”X3”; singles to 10 pack)	12.45-24.40
Bone Wax	4.04
Drill Items	63.00-350.00
Burrs (Round, Carbide, Fluted, Diamond)	63.00-350.00
Router (Straight, Tapered)	102.49-112.00
Perforator	182.00
Sutures and Needles	0.74-7.07
Nonabsorbable (Silk, Synthetic Monofilament, Braided Polymer)	0.74-7.07
Absorbable (Synthetic Braided, Synthetic Monofilament, Gut Plain and Treated, Synthetic 10-0)	1.26-5.46
Instruments	1.48-16.67
Blades (Beaver, Micro, Sickle, # 10, 11, 15)	1.48-16.67
Implants	431.88-3643.00
Onlays	
Nonsuturable Collagen (1”x1”-5”x5”)	432-1147.79
Suturable Collagen (1”x1”-5”x5”)	502.43-1114.79
Bovine Pericardium (6x8 cm)	431.88-580.84
Mesh (Medium to High-Density Polyethylene + Titanium, All Sizes)	424.00-3643.00
Aneurysm Clip (all Types)	418.00
Plating (Burr Hole Cover, Dogbone, Rectangle/Square)	53.00-286.00
Personal Protective Equipment	.69-3.50
Gowns (All Sizes, Types)	1.88-3.50
Gloves (All Sizes, Types)	0.69-1.52
Drains	301.02-1164.44
Catheters	
EVD + Collection System	301.02-798.00+ 466.44
Lumbar + Collection System	269.00-302.00 + 592.00
Surgical Drains	
Round	7.02
Bile Bag	5.69
Penrose	0.36

Hemostatic instruments (bipolars) and drill bits also varied significantly ($55.48-$537 and $63-$350, respectively). Other items' costs varied by orders of magnitude based on volume, including hemostatic matrices ($53.00 and $229.99). Sutures, however, varied little despite individual and pack prices ($0.74-$7.07).

**Figure 1:**
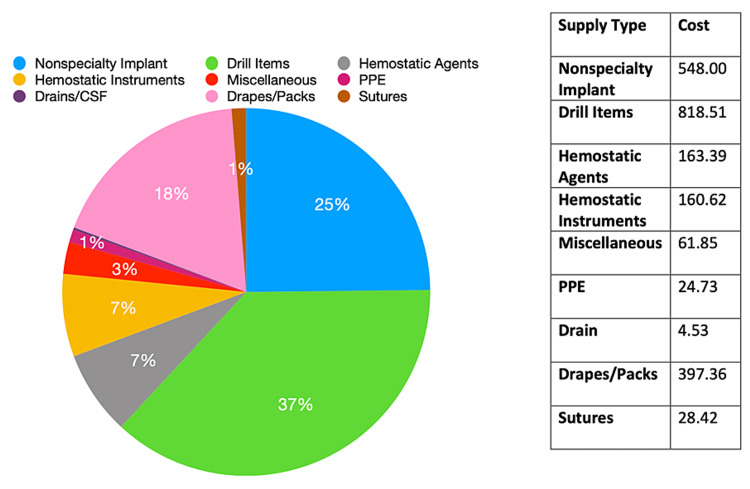
Extra-Axial Hematoma Evacuation Cost analysis of epidural hematoma evacuation with a total cost of $2207.41. The highest-costing category is Non-Specialty Implants. The lowest-costing categories are Sutures and PPE. PPE, personal protective equipment.

**Figure 2:**
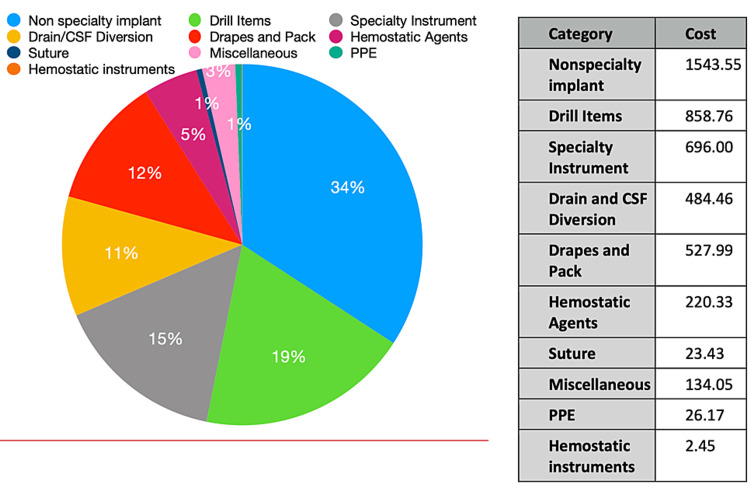
Left Craniotomy for Resection of Brain Lesion Single-use item cost data for a supratentorial parenchymal brain tumor resection costing a total of $5079.04. The highest costing category is Non-specialty Implants while the lowest costing categories are PPE, Sutures, and Hemostatic Instruments. PPE, personal protective equipment.

**Figure 3:**
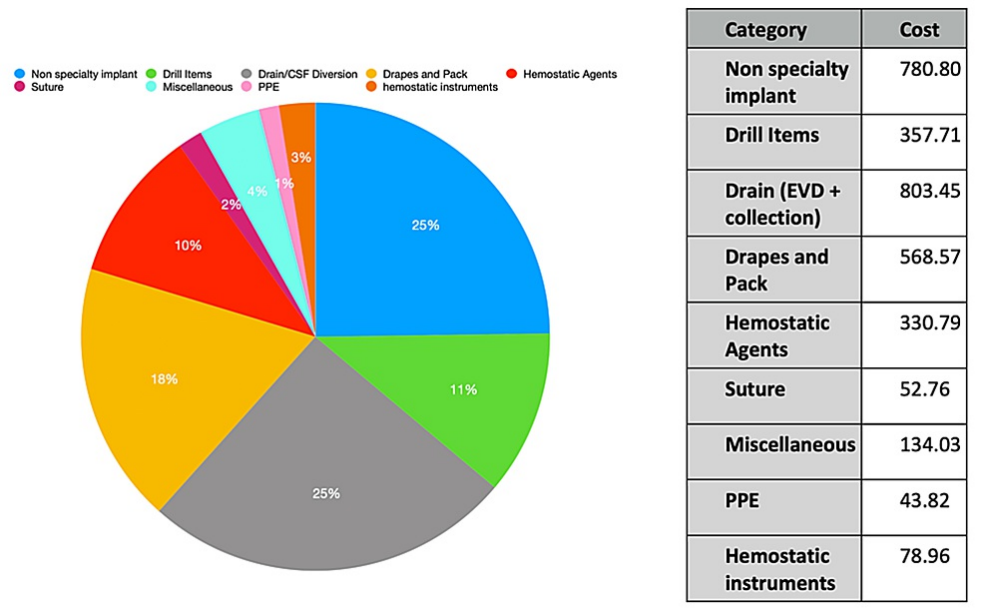
Suboccipital Craniectomy With Cervical 1 Laminectomy Single-use item cost data for a suboccipital craniectomy with a cervical laminectomy costing $3150.89. The highest costing category is Non-specialty Implants and Drains while the lowest costing category is PPE. PPE, personal protective equipment; EVD, external ventricular drain.

Case-based analysis revealed the following: an epidural hematoma evacuation cost $2207.41, following the overall database pattern with the non-specialty implant category contributing the most to the total expense and the PPE category contributing the least (Figure 1). A supratentorial brain tumor cost $5079.04 with the implants constituting the most expensive items and hemostatic instruments contributing the least (Figure 2). A suboccipital craniectomy with a cervical laminectomy cost $3150.89, with the drain category contributing the most to the total expense (external ventricular drain without collection system) and the PPE category contributing the least (Figure 3). 

## Discussion

The cost of many commonly used surgical items is staggeringly high [1-8]. Awareness of item costs, especially for those that are single-use and exchangeable, is prudent for economic stewardship in the operating room. 

Before our study, little research evaluated how using common surgical tools impacted the overall cost of typical neurosurgical procedures. For what literature does exist, evaluations look at item cost peripherally or in a manner lacking the necessary information and analysis for practitioners to apply such findings to their own practice [2-6]. For example, a study performed by Zygourakis et al. quantified the use and cost of disposed surgical supplies in neurosurgery procedures, highlighting operative waste [2]. Another study by McLaughlin et al. examined how the economic value of neurosurgical care could be optimized based on the implementation of quality improvement initiatives focused on cost [9]. These results, however, lack the granular details at the individual items level. 

Our results demonstrate that costs for similar items are wide-ranging with similarly broad variability in overall cost distribution by case. Taken by category, non-specialty implants were the most expensive items ($53-$3643) while PPE was the least ($1.88-3.50). These results hold based on both unit cost and this cost multiplied by units used per case. For example, within the non-specialty implant category, there was significant intra-item variation within dural onlays ($431.88-1147.79); however, these were mostly used one time per case for nearly all cranial cases. For those cases without onlays, surgeons used primary dural closure or buttressed the durotomy with a graft harvest (pericranium or fat) to nullify this cost. Other implants, such as aneurysm clips, demonstrated minimal cost variation ($418) and infrequent use outside of vascular cases. 

There exists a significant price range for certain items such as dural onlays. The reason for this is beyond the purpose of this article but is likely due to non-linear material/production costs prior to the item's arrival at our hospital. The contracted price for items is established prior to item use with negotiation performed at set intervals. 

Conversely, PPE was one of the more used categories, although contributing the least to the overall cost for all case types. As an example, the most expensive PPE item in our database is a gown costing $3.50. Therefore, despite common usage, it does not greatly contribute to the overall single-use item expense at our institution.

There were several similar items with variable costs associated with finer, more specialized versions. Bipolars, for example, ranged from $55.48 to $537.00 and drill bits ranged between $63.00 and $350.00. The more expensive version of the former was used in specialty vascular cases whereas the latter was used for complex skull base approaches. This variation reflects surgeon-guided preference, although these specific tools were not selected by all surgeons performing similar cases. 

Additional cost variation exists within items based solely on size and packs versus single distribution. For example, surgical patties become more expensive with size, which is then multiplied by pack quantity. For cases in which multiple patty sizes are necessary, it is wise to consider the quantity in which these are opened in order to avoid surgical waste and unnecessary costs. Hemostatic matrices followed similarly according to size variation ($53-229.99). In contrast, blades did not have a large intra-item cost range ($1.48-16.67). 

In our analysis of cost by case, the complexity of the surgical procedure ultimately guided the average cost of single-use items and how many items would be used. For an extra-axial hematoma evacuation at our institution (the lowest costing sample case at $2207.41), items cost $32.45/item whereas a craniotomy for intraparenchymal tumor (the highest costing sample case at $5079.04) cost $65.96/item. This seems intuitive, as the more involved and riskier the procedure is, the more likely items will be specialized (i.e. bipolars) and consumed (i.e. hemostatic agents). The importance of specifying case-by-case needs via preference card or careful conservation with the operating room staff is therefore essential to avoid open items over- or under-suited for the case requirements. 

There are several limitations to our study. First, costs are at least partially dependent on institutional agreements and vendors. However, the relationship between costs, grouped as in this study, seems to be similar to those reported elsewhere [1,3,4,9-13]. Second, this study only encapsulates a single institution within a 12-month period at which time the COVID-19 pandemic impacted medical supply chains [14]. This resulted in small, but measurable variations in some items, on a scale of cents to several dollars. When this occurred, the number of identical items with discrepant costs was evaluated, and the decision was made by two separate reviewers to either take an average of the two or the more represented cost of the two groups. Finally, surgeon preference within our institution is not reflected in our de-identified findings. The findings reported here are instead taken as a collective representation of a single institution. This methodology was chosen intentionally to match the realistic cost profile of an academic center where some degree of surgeon preference, and thus item variation, is permitted. Notably, all the items included in this review were frequently used. 

## Conclusions

While institution dependent, awareness of cost generators in craniotomy cases can help surgeons better choose tools based on need. Neurosurgeons should keep in mind alternatives to disposable items when feasible. Additionally, surgeons should be mindful of items that are inadvertently opened for cases for which they are not necessary and provide education to staff regarding instrument preferences to minimize exorbitant expenses.
